# Mate choice in the brain: species differ in how male traits ‘turn on’ gene expression in female brains

**DOI:** 10.1098/rspb.2024.0121

**Published:** 2024-07-31

**Authors:** Jason Keagy, Hans A. Hofmann, Janette W. Boughman

**Affiliations:** ^1^ Department of Ecosystem Science and Management, The Pennsylvania State University, University Park, PA 16802, USA; ^2^ Department of Integrative Biology, Michigan State University, East Lansing, MI 48824, USA; ^3^ Department of Integrative Biology, Institute for Neuroscience, The University of Texas at Austin, Austin, TX 78712, USA

**Keywords:** brain, gene expression, mate choice, speciation, stickleback

## Abstract

Mate choice plays a fundamental role in speciation, yet we know little about the molecular mechanisms that underpin this crucial decision-making process. Stickleback fish differentially adapted to limnetic and benthic habitats are reproductively isolated and females of each species use different male traits to evaluate prospective partners and reject heterospecific males. Here, we integrate behavioural data from a mate choice experiment with gene expression profiles from the brains of females actively deciding whether to mate. We find substantial gene expression variation between limnetic and benthic females, regardless of behavioural context, suggesting general divergence in constitutive gene expression patterns, corresponding to their genetic differentiation. Intriguingly, female gene co-expression modules covary with male display traits but in opposing directions for sympatric populations of the two species, suggesting male displays elicit a dynamic neurogenomic response that reflects known differences in female preferences. Furthermore, we confirm the role of numerous candidate genes previously implicated in female mate choice in other species, suggesting evolutionary tinkering with these conserved molecular processes to generate divergent mate preferences. Taken together, our study adds important new insights to our understanding of the molecular processes underlying female decision-making critical for generating sexual isolation and speciation.

## 1. Introduction

Choosing a mate is a key fitness decision [1] often crucial to speciation [2–4]. Much research has sought to identify the displays individuals assess when making mate choice decisions [5,6] as well as understand the evolution and divergence of mate preferences [4,7,8]. However, we know little about the cognitive and molecular mechanisms that underpin such decision-making or how these mechanisms vary across species [9,10]. Comparative transcriptomic studies, where constitutive gene expression profiles are systematically analysed across different populations, species and environments, have already provided important insights into the evolution of molecular mechanisms underlying various complex behaviours, such as learned vocalizations [11], mating systems [12,13], and cooperation [14]. Recent studies have also identified dynamic transcriptomic variation related to mate choice [15,16]. Yet whether and how the neuromolecular mechanisms reflecting these decision-making processes change as mate preferences diverge in the process of speciation has not been examined, nor do we know how variation in gene expression contributes to reproductive isolation. Given the importance of sexual selection to both evolution and speciation, this is a critical gap. To answer these questions, we need studies that examine gene expression differences in individual brains as they choose whether to mate with conspecifics or heterospecifics.

Neural transcriptomes exhibit both constitutive (e.g. dependent on species or sex) and dynamic (e.g. determined by behavioural state) components, which makes them uniquely suited to gain insight into the molecular processes underlying behavioural diversification and speciation [13,17,18]. We ask here how female brain gene expression varies among closely related species, focusing on transcriptomic responses during courtship in threespine stickleback fish (*Gasterosteus aculeatus*). The limnetic–benthic threespine stickleback species-pairs show parallel phenotypic divergence and speciation; each species has evolved independently in multiple lakes, showing repeated and substantially parallel divergence from ancestral marine fish in ecological and reproductive traits [19,20], confirming that both sexual selection and natural selection contribute [20–24]. Limnetic and benthic species experience strong sexual isolation, but mate freely with their own species whether from their own or another lake [22]. Moreover, prior research has revealed that the species have diverged in female preferences for nuptial colour, odour, courtship behaviour, and body shape and size; and females reject heterospecific males based on differences in these traits [23,25–29]. Finally, limnetic females are more responsive to male courtship and have stronger conspecific preferences than benthic females [26,30].

**Figure 1.**
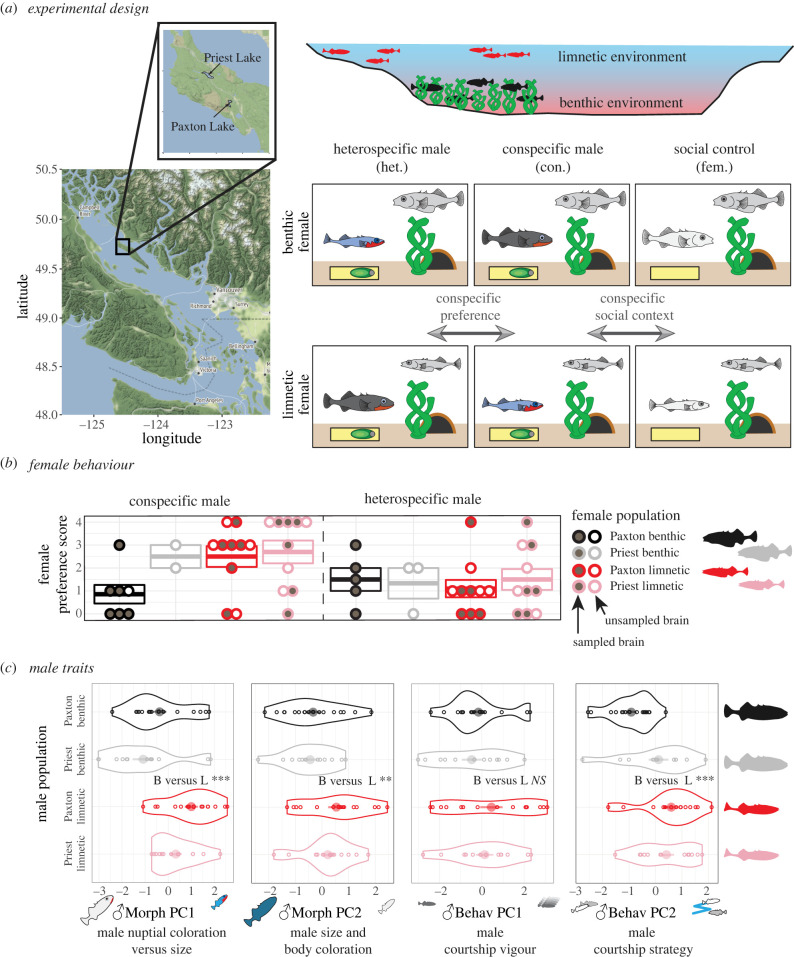
Study overview. (*a*) Experimental design. Male and female stickleback fish were sampled from two lakes on Texada Island, British Columbia and brought into the lab. Females (right top corner of each panel) were exposed to three treatments before they were euthanized and brains were removed: heterospecific male courtship (het.), conspecific male courtship (con.), or a conspecific female (fem.) from their home aquarium. (*b*) Female behaviour. Females showed stronger preference for conspecific males than for heterospecific males, especially if the female was limnetic. Boxes indicate means ± s.e. Each circle represents an individual female. Circles that are filled indicate females that were sampled for RNAseq. (*c*) Male traits. Five male morphological traits and five male behavioural traits were collapsed into two principal components (PCs) each to summarize male variation. Male morphological PCs differentiated limnetic (L) from benthic (B) fish (Welch two-sample *t*-test: Mmorph PC1: *t*_52.0_ = −4.13, *p* = 0.00013, Mmorph PC2: *t*_54.5_ = −2.76, *p* = 0.0078). The first male behavioural PC (courtship vigour) did not distinguish species (*t*_52.06_ = −1.39, *p* = 0.17), but the second one, which describes well known species differences in courtship strategy, did (*t*_54.73_ = −3.49, *p* = 0.00095).

In our study, limnetic and benthic females were collected from two lakes where the limnetic–benthic divergence is well studied and thought to be evolutionarily independent to provide two evolutionary replicates for testing parallel patterns of gene expression. Once in the laboratory, females were courted by either a conspecific or heterospecific male from their lake or were placed with a conspecific female from their lake as a social control (figure 1*a*). We quantified male morphological and behavioural display traits, female courtship behaviours, and female preference. Because the sample sizes were very small for one of the benthic populations, we generated whole-brain transcriptomes from females from two limnetic and one benthic population (see also Material and methods). Finally, we evaluated the relationship of these neural transcriptomes with behavioural and morphological data from the behavioural experiment. Although our transcriptome data ended up being unbalanced, we can still compare limnetic populations with each other to test for parallel changes among them and we can compare limnetic and benthic populations from the same lake to test for divergence in gene expression as part of the speciation process.

We make the following predictions for our transcriptome data. First, given the divergent evolution of benthic and limnetic species in both ecology and reproduction, we predict divergence between the species in constitutive gene expression patterns. Second, we predict the two limnetic populations will show similar patterns of constitutive gene expression because of their parallel evolution in different lakes. Third, we predict females will show dynamic brain gene expression differences depending on the social context (conspecific courtship, heterospecific courtship, or social control) as they will not only be experiencing different stimuli but also making different decisions with respect to reproduction. Given that benthic and limnetic species have divergent preferences and are influenced by different male traits, we predict that the genes implicated in conspecific preference and social context might vary, especially when comparing benthic and limnetic populations from the same lake, where reinforcement is expected to have occurred. Alternatively, these dynamic attributes of the brain transcriptome may be similar between benthic and limnetic species owing to being dependent on conserved molecular pathways. Likewise, we predict the two limnetic populations will show similar patterns of dynamic gene expression as a result of parallel evolution in the two different lakes. Alternatively, the parallel evolution of behaviour may not be underpinned by parallel genomic or transcriptomic processes [31–33].

Finally, we predict that gene expression in female brains will respond to variation in male displays in a quantitative manner. What we envision here is that gene expression levels will increase (or decrease) in a linear manner when the display is more attractive, and the opposite will occur when the display is disliked. Because females of the two species either accept or reject males based on distinct display traits, their gene expression responses should be in opposing directions, in other words, up- or down-regulated in proportion to the trait value experienced. Another possibility is that different traits might activate similar gene expression patterns, with a given trait only activating expression in the relevant species that uses that trait for mate choice. Both possibilities would be reflected by a significant species-by-trait interaction in linear model analysis. The absolute direction of response for each species is difficult to predict because changes in gene expression may ultimately lead to activation or inhibition of behaviour. Some of the strongest evidence linking female mating decisions to male displays and reproductive isolation in a neuromolecular manner would be to find differential gene expression patterns that are associated with *both* female choice behaviour and male display as this would imply they are connected mechanistically.

## 2. Material and methods

Benthic and limnetic threespine stickleback were collected from two lakes on Texada Island, British Columbia (figure 1) and transferred to Michigan State University. Additional fish care information is in the electronic supplemental material.

### (a) Behavioural trials

We used standard methods for assessing female mate preference in stickleback, in which a female is courted by a single male [25,26,34]. Each female subject experienced one of three trial conditions: (1) courtship by a male from their own lake and species (conspecific courtship, *n* = 29), (2) courtship by a male from their own lake and the opposite species (heterospecific courtship, *n* = 28), or (3) control in which female subjects were with another female from the same lake and species (social control, *n* = 26) (figure 1*a*).

During May–August 2014, reproductive males were taken from holding aquaria and placed individually in new visually isolated aquaria with nesting materials and enticed to build a nest (see electronic supplementary material for more details). All behavioural trials commenced between 09.30 ET and 12.30 ET. We verified each female's reproductive status by gently squeezing her abdomen to confirm presence of ripe eggs [27]. For the courtship treatments, females were placed in an opaque holding container just below the water surface in the male's nesting aquarium for a 5 min acclimation period. The female was then remotely released, and the behaviours of both male and female were recorded using the event recorder JWatcher 1.0 (http://www.jwatcher.ucla.edu/) by two observers. The social control treatment was done similarly except that the stimulus fish was a non-reproductive female from the subject's home aquarium.

After 20 min or spawning (whichever came first, see electronic supplementary material for more details), the female subject was killed by rapid cervical dissection, and the brain was immediately dissected under a microscope in a Sylgaard 184-lined Petri-dish filled with Ringer's solution. Brains were stored overnight in RNAlater at 4°C and then transferred to −20°C until RNA extraction.

### (b) RNA-seq

In September 2014, brains were shipped on dry ice to The University of Texas at Austin (UT Austin), where RNA was extracted using the Maxwell 16 LEV simplyRNA Tissue Kit, which uses a robot to increase consistency. At the UT Austin Genomic Sequencing and Analysis Facility (GSAF) each sample (all with RNA integrity number (RIN) > 7.8, 8.8 ± 0.4 mean ± s.d., 11 samples do not have RIN scores) was prepared for RNA-seq using poly­A mRNA capture and given a unique barcode. A single library of all multiplexed samples was sequenced across eight lanes of an Illumina Hiseq 2500 with 2 × 50 paired-end (PE) chemistry. Sample size for each treatment for Paxton limnetic, Paxton benthic, and Priest limnetic populations was *n* = 5 or 6. Brains were randomly chosen for each population and treatment if there were more than six to choose from. Very few Priest benthic females became reproductive, resulting in three or fewer brains per treatment for this population. Given that we were expecting large individual variation in transcriptomes, we focused our sequencing effort on the other three populations with larger sample sizes. Bioinformatic analyses were carried out using the computational resources of the Texas Advanced Computing Center (TACC).

RNA-seq resulted in 34.2 ± 4.6 million reads (mean ± s.d.) per sample per sequencing direction (forward or reverse). Reads from all eight lanes were combined for each uniquely barcoded individual, separately for forward and reverse reads. We conducted quality control checks using *fastqc* (v. 0.11.1). We then ran *Trimmomatic* (v. 0.33) to remove a small amount of adapter contamination. Next, reads were aligned to the *Gasterosteus aculeatus* Ensembl BROAD S1 draft genome (version 78) using *bwa* (v. 0.7.7). We obtained expression information for 21 798 genes (electronic supplementary material, table S1). *Samtools* (v. 1.2) was used to convert sam to bam files, and then sort and index them. These sorted and indexed bam files were then passed to *bedtools* (v. 2.23.0) to count gene transcripts. The resulting gene counts were analysed quantitatively using *DESeq2* [35] and *WGCNA* [36,37] in R v. 4.2.2 [38] as described below.

### (c) Analysis

We derived two principal components (PCs) each for variation in female behaviour (Fbehav), male behaviour (Mbehav) and male morphology (Mmorph) to facilitate the integration of transcriptome data with female mating behaviour and the male traits females experienced (electronic supplementary material, figure S1). Derivation and analyses of female and male phenotypic data are described in the electronic supplementary material.

**Figure 2.**
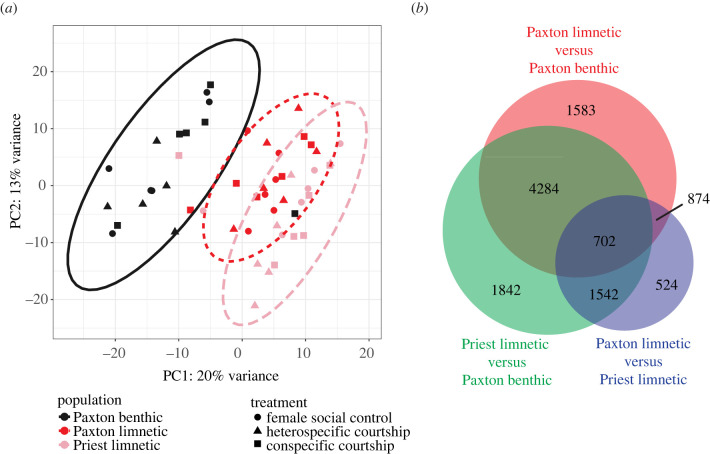
Species have highly divergent gene expression patterns. (*a*) Principal components analysis (PCA) of variance-stabilized normalized counts of 90% most variable genes. PC1 clearly separates populations in expected ways. For example, limnetic populations overlap and are very well separated from the benthic population. Ovals are 95% confidence ellipses. Two samples appeared to have been swapped (Priest limnetic and Paxton benthic; each is in the centre of the other's population distribution) and are not included in further analyses. (*b*) Venn diagram comparing overlap of population comparison differentially expressed genes (false dicovery rate (FDR) corrected *p*-value <0.1). Again, there were many more genes that distinguished benthic from limnetic fish than limnetic fish from different lakes.

Using the *rlog* function in the *DESeq2* library [35] and a design matrix with an intercept only, we first applied a ‘regularized log’ transformation to the gene expression count matrix that was exported for the weighted gene co-expression network analysis (WGCNA) and candidate gene analyses (electronic supplementary material, table S10, more below). Using these normalized and variance-stabilized gene expression counts, we conducted a principal components analysis (PCA) on the 90% most variable genes as an initial step to visualize how populations and treatments separate along the axes of greatest gene expression variation. Two samples appeared to have been swapped and were removed from analyses (figure 2*a*). The original untransformed gene expression count matrix was then analysed using standard methods [35] with a model where gene expression counts were predicted by the independent effects of treatment and population. To test for constitutive gene expression differences between populations, subsequent contrasts were computed using the ‘normal’ shrinkage option and an *α* of 0.1. To determine genes involved in conspecific preference or social context, treatment difference contrasts were calculated separately for each population from a second model in which gene expression counts were predicted by the interactive effect of treatment and population. The *gprofiler2* library was used for gene ontology (GO) analysis to identify the biological processes, cellular locations, and molecular functions associated with differentially expressed genes [39].

To identify gene modules that may underpin shared biological functions, we conducted WGCNA using standard methods for signed network construction using the *WGCNA* library [36,37]. Briefly, we used the 90% most variable genes from the normalized and variance-stabilized gene expression count matrix (without the two samples suspected of having been swapped). Additional quality control steps eliminated two samples that had the lowest read-mapping percentage. Using the TACC high-performance computing cluster, we tried different combinations of parameters to optimize network construction rather than simply relying on defaults. The analysis presented here used the following parameters: maxBlockSize = 30 000, power = 9, network-type = ‘signed’, corType = ‘bicor’, maxPOutliers = 0.05, minModuleSize = 30, mergeCutHeight = 0.25, deepSplit = 2 (default), and detectCutHeight = 0.995 (default). Modules were analysed first using *t*-tests to compare sympatric species (Paxton limnetic versus Paxton benthic), limnetic populations from different lakes (Paxton limnetic versus Priest limnetic), and treatments (separately for each population). Then we constructed linear models (using the *lm* function in the *stats* library [38]) with module eigengene expression predicted by population, morphological or behavioural trait, and their interaction. We assessed the statistical significance of main and interaction effects using an empirical false discovery rate (eFDR) procedure [40]. Briefly, significance was initially calculated using an analysis of variance (ANOVA) table with type-II sums of squares from the linear model results (using the *Anova* function in the *car* library [41]). Then the gene expression counts were shuffled among samples 10 000 times and new significance values calculated each time. The eFDR was the proportion of times the true significance value was lower than or equal to the significance values calculated from these shuffled datasets. The *gprofiler2* library was used for GO analysis to identify the biological processes, cellular locations and molecular functions associated with different modules [39].

We also compiled a list of candidate genes from the existing literature that had previously been implicated in mate choice, and analysed their expression using linear models with gene expression predicted by population, morphological or behavioural trait, and their interaction and significance assessed using an eFDR procedure.

## 3. Results and discussion

### (a) Females preferred conspecific males and males differed in species-specific ways

Consistent with earlier work [22,26,42], females displayed a stronger behavioural preference for conspecific males than heterospecific males (ANOVA: *F*_1,49_ = 11.11, *p* = 0.016, figure 1*b*). Also consistent with earlier work, limnetic females showed especially strong conspecific preference (ANOVA: *F*_1,36_ = 16.90, *p* = 0.0062). Moreover, male morphological and behavioural display traits differed between the species (figure 1*c*).

### (b) Species show highly differentiated gene expression for individual genes

We first asked whether constitutive variation in female brain transcriptomes is concordant with previously described genetic differences between benthic and limnetic species [19,43–46]. We found that gene expression patterns were indeed highly differentiated between the one benthic population and two limnetic populations (figure 2; electronic supplementary material, figure S2). Unexpectedly, benthic and limnetic populations from the same lake had fewer differentially expressed genes (DEGs) than benthic and limnetic populations from different lakes (7443 (34% total genes) versus 8370 (38%), adjusted *p*-value <0.1, 21 796 total genes; figure 2*b*; electronic supplementary material, table S2), possibly owing to lake-specific ecology or low levels of past or contemporary gene flow [30] partly homogenizing genetic differences underlying constitutive expression patterns. The two limnetic populations had similar overall expression patterns (more overlap in scores of the first PC describing multivariate gene expression variation and fewer DEGs, figure 2), consistent with previously described parallel evolution [22,47,48]. Nonetheless, female brains of the two limnetic populations did show differences in gene expression (3 642 (17%) DEGs, adjusted *p*-value <0.1, 21 796 total genes; figure 2*b*; electronic supplementary material, table S2) and different gene expression patterns in response to specific male traits (see below).

### (c) Magnitude of gene expression differences between treatments reflects species differences in strength of courtship responsiveness and conspecific preference

**Figure 3.**
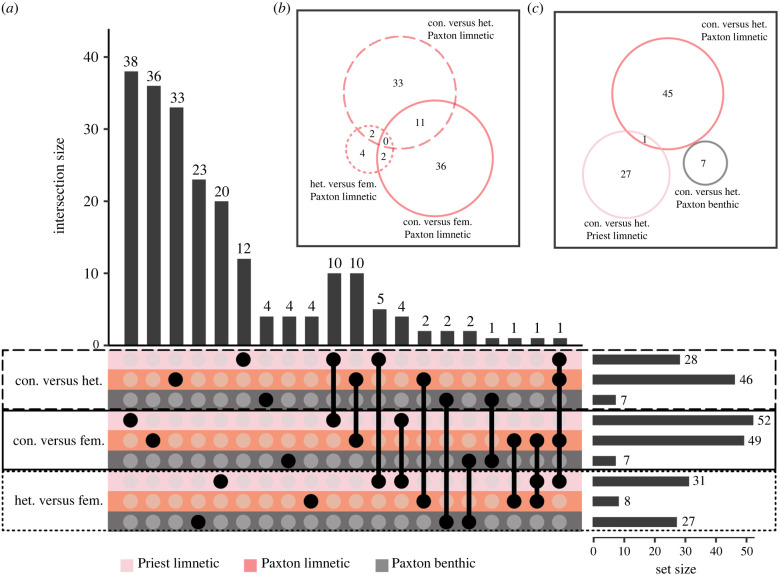
Differentially expressed genes from treatment comparisons. (*a*) UpSet plot showing all treatment comparisons separated by population. Solid circles connected by lines indicate the intersection between those sets—circles that are unconnected to any others indicate genes unique to that set. Generally, there is very little overlap between sets; when there is, it is between different comparisons within the same population or species. We used a significance threshold of false discovery rate (FDR) corrected *p* < 0.1. Traditional Venn diagrams can be derived from the UpSet plot as seen in (*b*) Paxton limnetic treatment comparisons and (*c*) conspecific versus heterospecific comparisons.

We euthanized all fish within 20 min of stimulus onset to reveal dynamic transcriptome activity during decision-making. Even with this short stimulus time, limnetic females from both lakes showed numerous DEGs in comparisons between the conspecific male treatment versus either the conspecific female (social control) or heterospecific male treatments (28–52 (0.13–0.24%), adjusted *p*-value <0.1, 21 796 total genes; figure 3; electronic supplementary material, table S3). Benthic females, in contrast, had fewer DEGs for both of these comparisons (7 (0.03%), figure 3), consistent with prior behavioural work indicating limnetic females are more responsive to male courtship and have stronger conspecific preferences than benthic females [26,48]. These patterns are interesting in light of earlier findings in the Panuco swordtail (*Xiphophorus nigrensis*), where females strongly prefer large courting males to small coercive males [49]. This robust behavioural preference in swordtails was reflected in the brain transcriptome: the brains of females in this choice situation have many more DEGs than females exposed to only small males or only females [15]. Recent work in another poecilid species, the guppy (*Poecilia reticulata*), also demonstrated that females that expressed a strong preference for colourful over drab males had many more DEGs than females that did not have a male coloration preference [16]. Taken together with our findings, these results point to stronger gene expression response with greater mate preference.

### (d) Genes involved in conspecific preference are population-specific

DEGs from the comparison of conspecific male with heterospecific male treatments (‘conspecific preference’, 7–46 DEGs (0.03–0.21%), adjusted *p*-value <0.1, 21 796 total genes; figure 3*c*; electronic supplementary material, table S3) support the conclusion that female brains are differentiating conspecific from heterospecific males and that dynamic changes in gene expression are involved in this decision-making. Notably, however, we found virtually no overlap in DEGs between populations for conspecific preference (figure 3*c*), suggesting that different genes are involved in conspecific preference in each population. We expected this for benthic compared with limnetic populations because they have distinct mating preferences and social behaviour [26,48,50]; our findings support that prediction. However, we expected some overlap between limnetic populations because of their parallel evolution in ecological and reproductive traits [26,48,50] and because of their overall similarity in constitutive gene expression discussed above. Instead, the little overlap between limnetic populations for conspecific preference is consistent with independent behavioural evolution in each lake [19] resulting in distinct molecular networks related to conspecific preference (reflected also by non-overlap of Gene Ontology (GO) terms; electronic supplementary material, table S4). This could have been either owing to the available genetic variants differing (*sensu* the mutation order hypothesis [51–53]), or because selection was less similar than thought. Although there are exciting examples of parallel gene expression changes underlying the parallel evolution of complex behavioural phenotypes [11–14,33], it seems that parallel phenotypic evolution often rests on only partly parallel genetic mechanisms [31,54], as is suggested by the data from the two limnetic populations here.

### (e) Gene co-expression network analysis supports individual gene analysis results

**Figure 4.**
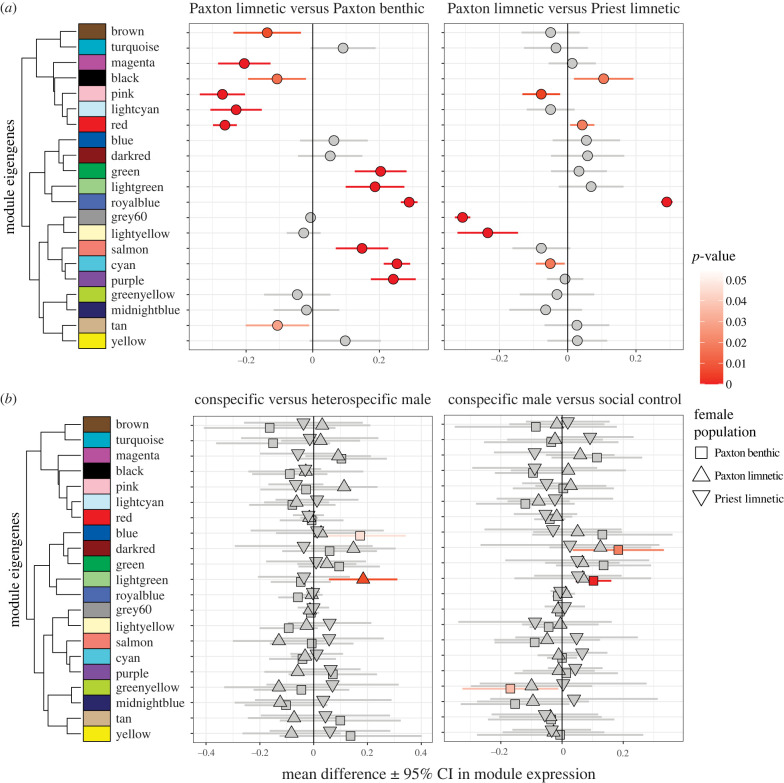
Comparisons of module expression between (*a*) populations and (*b*) treatments. Symbols and lines correspond to mean ± 95% confidence intervals for *t*-tests. Significant differences are indicated in colour.

We next used weighted gene co-expression network analysis (WGCNA [36,37]) to identify gene modules that may underpin shared biological functions (electronic supplementary material, figure S3 and table S5); expression of genes in a module can be summarized by eigengenes, the first PC of a given module. We identified 13 differentially expressed module eigengenes (DEMEGs) between limnetic and benthic populations from the same lake (figure 4; electronic supplementary material, table S6), similar to patterns we uncovered with the individual gene analyses. Also similarly, we found fewer DEMEGs when comparing limnetic populations from different lakes (7 DEMEGs; figure 4; electronic supplementary material, table S6). However, five of these seven modules also differentiate Paxton limnetic from Paxton benthic fish and so separate populations generally. Thus, we find support for our prediction of stronger divergence in constitutive gene expression between diverged species and weaker divergence between populations of limnetic fish that have evolved in parallel.

Although we found relatively few DEMEGs between treatments (figure 4; electronic supplementary material, table S6), we did identify two DEMEGs when females were courted by a conspecific versus heterospecific male; one for Paxton benthic and one for Paxton limnetic females. We suggest that these modules are involved in conspecific preference and may play an important role in sexual isolation. We also found three DEMEGs when benthic females were courted by a conspecific male versus interacted with a female, suggesting these modules are involved in evaluating males. We were surprised that benthic females showed a strong effect here as they neither exhibited strong preference nor strongly discriminated between males at the behavioural level (figure 1*b*) and they had the fewest DEGs for these comparisons (figure 3; electronic supplementary material, table S3). Module eigengene expression likely reflects female perception and decision-making rather than a final decision given the relatively short time (≤20 min) between trial start and sampling brains. We, therefore, examined next how female module eigengene expression relates to male display trait variation.

### (f) Module eigengene expression in the female brain reflects individual variation in a male sexually selected trait in population-specific ways

It is well established that variation in male traits influences both current and future female mating decisions [1,5,6,34], the specific traits that females focus on vary between populations [55,56], and changes in the expression and topology of gene networks interface with neural networks to influence future behaviour [57]. We, therefore, predicted that module eigengene expression in the female brain should vary dynamically in conjunction with male displays, likely in a species- or population-dependent fashion. Indeed, this was true for an important male morphological trait, throat colour, which varies between benthic and limnetic species, is involved in female choice and male competition differently for the two species, and is critical for sexual isolation [23,24,58–60]. Variation in male throat colour significantly predicted variation in the eigengene expression of two modules in a population-dependent manner (electronic supplementary material, figure S4 and table S7), with mean expression and slopes differing between the benthic population and at least one limnetic population, especially the sympatric one. Thus, the activity of brain gene co-expression modules was dynamically altered in female brains in response to this key male trait known to be subject to sexual selection and involved in sexual isolation. Importantly, the relationship between module eigengene expression and male throat colour was strongly divergent in benthic and limnetic females from the same lake, in parallel with strongly divergent preferences for this trait in these same females [23]. This finding argues that the differential recruitment of gene networks implied by these co-expression modules underpins divergence in mating behaviour and preference, thus contributing to reproductive isolation between diverging species.

Our finding that module eigengene expression responds to male throat colour helps to explain the low number of DEMEGs for treatment comparisons. Based on these results, females in the same treatment would be expected to have variable expression of some modules based on the trait values of the specific males courting them. Furthermore, although limnetic males tend to have more throat coloration than benthic males, there is overlap in the distribution, leading to imperfect correspondence between coloration and treatment, likely obscuring the gene expression signal from treatment comparisons.

### (g) Moving from individual traits to multivariate descriptions of male and female phenotypes

**Figure 5.**
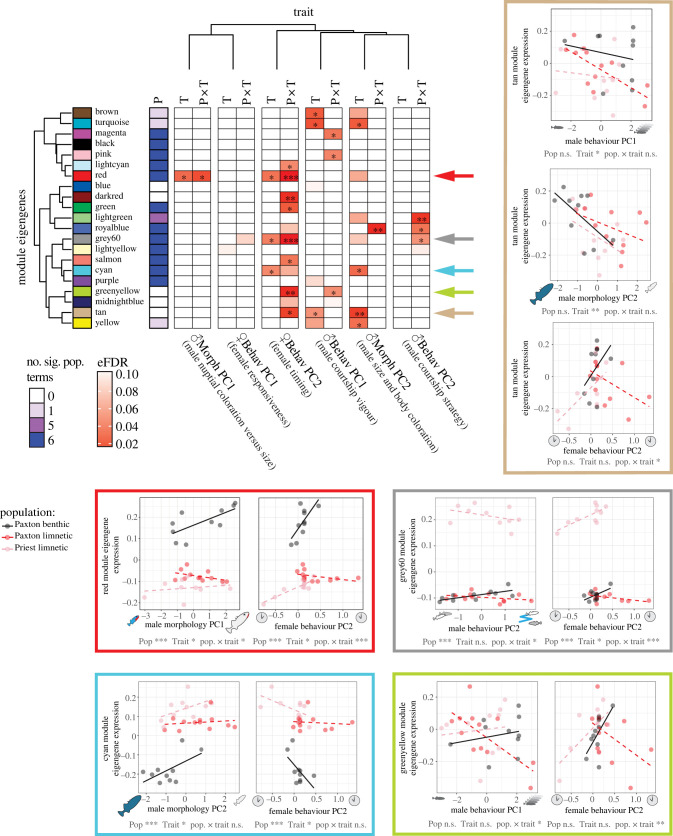
Linear models describing relationship between module eigengene expression and population, traits, and their interaction. Arrows indicate modules that respond to both a male display trait and female courtship behaviour; these relationships are further explored in scatterplots. ‘P’ = population term, ‘T’ = trait term, ‘P × T’ = population-by-trait interaction term with significance indicated: *** <0.001, ** <0.01, * <0.05. To focus on the trait and population-by-trait interaction effects, we have reduced the population term to a single column indicating in how many of the six models it was significant (at *p* < 0.05).

Mate choice decisions are typically based on more than a single trait [10,55,61,62]. We therefore used the axes of variation in male behaviour (Mbehav PC1 and PC2) and morphology (Mmorph PC1 and PC2) as well as female behaviour (Fbehav PC1 and PC2) we previously inferred using PCA (electronic supplementary material, figure S1) to integrate variation in these traits with our gene expression data. We did this using linear models, testing for population and trait PC main effects as well as their interactive effects on module eigengene expression (figure 5; electronic supplementary material, table S7). Significant main effects of trait PCs (with no population-by-trait interaction) indicate modules that respond to specific trait PCs in a consistent manner across all populations. Genes in these modules could reflect conserved patterns of gene expression during courtship. We did find this pattern with the PC describing male size and body coloration (Mmorph PC2) for four modules, the PC describing male courtship vigour (Mbehav PC1) for three modules, and the PC describing the nature of female response (Fbehav PC2) for one module, for a total of five unique modules. Several of these modules have clearly differentiated biological functions, according to GO analysis (electronic supplementary material, table S8), that suggest involvement of stress, metabolism, neural function and DNA/RNA processing molecular pathways.

Most importantly, however, significant population-by-trait interactions address our primary prediction that divergent selection on female mate preferences would result in divergent brain gene expression patterns in response to male display trait values, especially in benthic and limnetic females from the same lake. Indeed, we saw many modules with this pattern; seven modules showed a significant population-by-trait interaction and, in all cases, limnetic and benthic populations from the same lake had opposing slopes (figure 5). When we shifted our focus to female behaviour in response to courtship, we found a similarly large number of modules (*n* = 8) that showed a population-by-trait interaction, specifically the nature of female response (early versus late, Fbehav PC2). Once again, we observed that limnetic and benthic populations from the same lake had opposite slopes, showing strong divergence in gene expression in association with female behaviour.

GO analysis of the eight modules with a significant population-by-Fbehav-PC2 interaction indicates distinct biological functions being overrepresented by module genes (electronic supplementary material, figure S5 and table S8), especially for the greenyellow and green modules: DNA/RNA processing, metabolic processes, and cellular stress response (greenyellow) and synaptic signalling, ion transport, cell–cell signalling and neural development (green). Clearly something very different is happening in female brains of sympatric species of stickleback. The preponderance of genes with neural functions (green module) and those influencing future gene expression and response to stress (greenyellow module) suggests those modules are mediating activity in the brain involved in decision-making. The different expression patterns for limnetic and benthic females from the same lake point to a key role for these modules in isolating the species.

Even more interesting, for five modules there was overlap where a module's eigengene expression was associated with *both* male trait and female behavioural variation (figure 5). We interpret this to indicate that the male trait elicits a specific neurogenomic response in the female brain that, in turn, influences female choice behaviour. For example, the red module showed lower eigengene expression in Paxton limnetic females when they were courted by males that they were expected to find more attractive (smaller and with brighter blue eyes and more extensive red throat coloration). These females in turn had lower eigengene expression of the red module if they showed more interest later in courtship. Paxton benthic females showed the exact opposite relationships between red module eigengene expression and male morphology and female behaviour. The finding of overlap in modules showing associations with male traits and female behaviour suggests these modules are important for premating isolation.

### (h) Candidate gene expression is also associated with trait values

**Figure 6.**
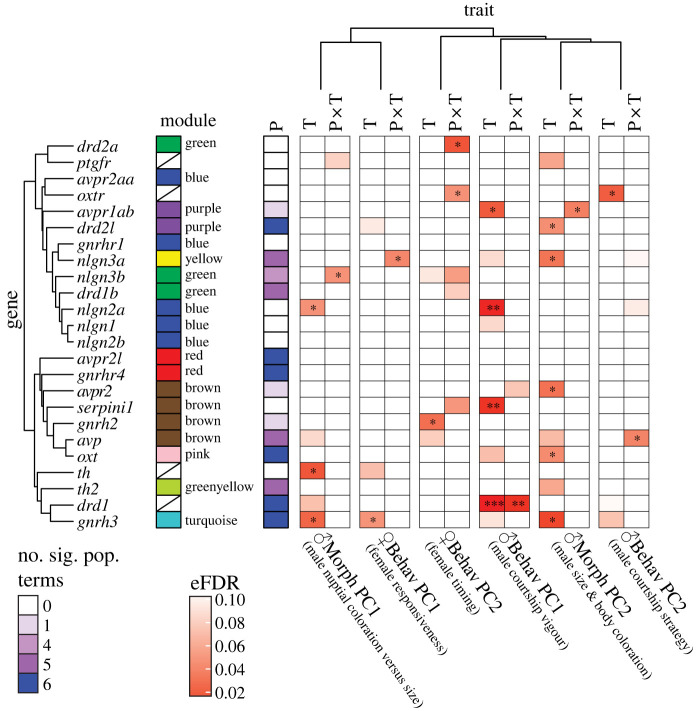
Linear models describing relationship between candidate gene expression and population, traits, and their interaction. ‘P’ = population term, ‘T’ = trait term, ‘P × T’ = population-by-trait interaction term, with significance indicated: *** <0.001, ** <0.01, * <0.05. To focus on the trait and population-by-trait interaction effects, we have reduced the population term to a single column indicating in how many of the six models it was significant (at *p* < 0.05).

Numerous candidate genes have previously been implicated as playing a role in female mate choice and social decision-making more generally [9,63]. We therefore mined our transcriptome dataset for candidate genes representing five distinct and well studied neuroendocrine and neuromodulatory pathways. Specifically, we focused on genes involved in (1) **gonadotropin-releasing hormone (GnRH) signalling**, owing to their role in reproduction [64]: *gnrh2*, *gnrh3*,* gnrhr1*, *gnrhr4*; (2) **nonapeptide signalling**, known for regulating affiliative behaviour [65]: *avp*, *avpr1ab*,* avpr2*, *avpr2aa*, *avpr2l*,* oxt*, *oxtr*; (3) **dopamine signalling**, which is important for motivational processing [66]: *th*, *th2*, *drd1*, *drd1b*, *drd2a*, *drd2l*; (4) **prostaglandin F2 alpha (PGF2α) signalling**, as the ovarian hormone PGF2α is a well known regulator of reproductive behaviour in fishes [67,68]: *ptgfr*; and (5) specific genes important to **synaptic plasticity** that have previously been implicated in mate choice decisions in poecilid fishes [15,16,69]: *nlgn1*, *nlgn2a*, *nlgn2b*, *nlgn3a*, *nlgn3b*, *neuroserpin1 *(*serpini1*). We found that 20 of these 24 genes (83%) were members of nine different gene co-expression modules (figure 6), with the blue (5 genes) and brown (4 genes) modules most prominently represented (recall that the brown module was correlated with Mbehav PC1, courtship vigour). We then used linear models as with the gene co-expression modules above to discover that 14 genes (58%), representing four of these pathways (all except for PGF2α signalling), showed significant differences in expression between populations (figure 6, electronic supplementary material, table S9).

When we focused on significant relationships between candidate gene expression and specific male or female traits, we discovered several intriguing associations. For example, the expression of *gnrh3*, which has previously been shown to gate mating preferences in medaka (Japanese rice fish, *Oryzias latipes*) [70], was associated with female responsiveness (Fbehav PC1), while *gnrh2* expression reflected female timing (Fbehav PC2). Note that GnRH2 plays a critical role in the integration of energy homeostasis and sexual behaviour in mammals and teleosts [71,72]. Looking at the behaviour and morphology of the males, we found that dopaminergic and nonapeptide signalling along with synaptic plasticity in the female brain reflect male courtship vigour (Mbehav PC1), while oxytocin receptor expression is related to male courtship strategy (Mbehav PC2). In addition, GnRH and dopaminergic signalling along with synaptic plasticity reflect a male's nuptial coloration (Mmorph PC1) and male size and body coloration (Mmorph PC2), with nonapeptide signalling also associated with Mmorph PC2. Taken together, our candidate gene analysis is consistent with findings in other species.

## 4. Conclusion

Despite their close evolutionary relationship, we find substantial differences in gene expression for limnetic and benthic females making mate choice decisions. This differentiation is not due simply to differences in magnitude of expression in the brains of the two species. Indeed, our most novel finding is that brain gene expression responds to male display traits in opposite directions for the two sympatric species, mirroring contrasting female behavioural responses to those displays that are known to contribute to sexual isolation. Male displays that trigger elevated expression of a module in female brains for one species trigger reduced expression in the other sympatric species. We find support of not only our expectation of constitutive expression differences in the brains of benthic and limnetic fish, but also our key prediction of differential expression driven by diverged female preferences for diverged male display traits in a quantitative manner related directly to female experiences during courtship. Taken together, our results provide novel insights into the neuromolecular processes that govern reproductive isolation of diverging species.

## Data Availability

Data and analysis code are available from the Dryad Digital Repository [73]: https://doi.org/10.5061/dryad.ncjsxkt3g. Raw sequencing data are deposited in NCBI Gene Expression Omnibus (GEO) Ascension GSE263554. Data for analysis are also provided in the electronic supplementary material [74].
